# p16^INK4a^ expression in retinoblastoma: a marker of differentiation grade

**DOI:** 10.1186/s13000-014-0180-1

**Published:** 2014-12-11

**Authors:** Yue Liu, Xiufeng Zhong, Shangtao Wan, Wenxin Zhang, Jianxian Lin, Ping Zhang, Yongping Li

**Affiliations:** State Key Laboratory of Ophthalmology, Zhongshan Ophthalmic Center, Sun Yat-Sen University, 54 S Xianlie Rd, Guangzhou, 510060 China

**Keywords:** Retinoblastoma, Differentiation, p16^INK4a^, Immunohistochemistry

## Abstract

**Background:**

The tumor suppressor protein p16^INK4a^ has been extensively studied in many tumors with very different results, ranging from its loss to its clear overexpression, which may be associated with degree of tumor differentiation and prognosis. However, its expression remains unclear in human retinoblastoma (RB), a common malignant tumor of retina in childhood. The aim of this study was to explore the expression pattern of p16^INK4a^ in RB, and the correlation between p16^INK4a^ expression and histopathological features of RB.

**Methods:**

Sixty-five cases of RB were retrospectively analyzed. Paraffin-embedded blocks were retrieved from the archives of ocular pathology department at Zhongshan Ophthalmic Center of Sun Yat-sen University, China. Serial sections were cut and subjected to hematoxylin and eosin staining. Immunohistochemical staining was further done with antibodies p16^INK4a^, CRX and Ki67. The correlation of p16 ^INK4a^ expression with CRX and Ki67 and clinicopathological features of RB were analyzed.

**Results:**

RB tumor histologically consists of various differentiation components including undifferentiated (UD) cells, Homer-Wright rosettes (HWR) or Flexner-Winterstein rosettes (FWR) and fleurettes characteristic of photoreceptor differentiation or Retinocytoma (RC). p16^INK4a^ expression was negative in both fleurette region and the residual retinal tissue adjacent to the tumor, weakly to moderately positive in FWR, strongly positive in both HWR and UD region. However, CRX had the reverse expression patterns in comparison with p16^INK4a^. It was strongly positive in photoreceptor cells within the residual retina and fleurettes, but weakly to moderately positive in UD area. Together with Ki67 staining, high p16^INK4a^ expression was associated with poor histological differentiation of RB tumors, which had higher risk features with the optic nerve invasion and uveal invasion.

**Conclusions:**

p16^INK4a^ expression increased with the decreasing level of cell differentiation of RBs. RB tumors extensively expressing p16^INK4a^ tended to have higher risk features with poor prognosis. This study suggested that p16^INK4a^ would be a valuable molecular marker of RB to distinguish its histological phenotypes and to serve as a predictor of its prognosis.

**Virtual Slides:**

The virtual slide(s) for this article can be found here: http://www.diagnosticpathology.diagnomx.eu/vs/13000_2014_180

## Background

Traditionally, level of neoplastic differentiation is a crucial index of adjuvant chemotherapy and prognosis. Retinoblastoma (RB), the most common primary malignant intraocular tumor in childhood, has different pathological phenotypes from undifferentiated tumor cells, Homer-Wright rosettes (HWR) or Flexner-Winterstein rosettes(FWR) to fleurettes. These phenotypes reflect to some extent the level of tumor differentiation which is a key factor in grade of retinoblastoma and prediction of its prognosis [1,2]. For example, retinocytoma (RC), also called retinoma, has been proposed to be the benign variant of RB since it is largely composed of benign-appearing cells with elongated eosinophilic fleurettes similar to the inner segment of photoreceptor cells and because it has better prognosis than RB in general [3-5]. However, these components with various degree of differentiation have been a challenge to be morphologically identified by researchers, sometimes even by experienced pathologists. It would therefore be very useful to find out a panel of molecular markers to distinguish them and to predict progression of RB.

p16^INK4a^, a tumor suppressor protein, has played an important role in cell cycle control, cell senescence and tumor development. It has been extensively studied in both benign and malignant lesions with very different results, ranging from its loss to its clear overexpression [6]. For instance, in benign lesions such as nevi and neurofibroma, p16^INK4a^ overexpression was associated with senescence [7,8]; whereas in malignancy such as HPV-positive cervical cancer, breast cancer and colorectal adenocarcinoma, it appeared to be associated with high-grade tumors along with RB gene alterations [9-11]. In recent years, p16^INK4a^ has been used as a diagnostic marker to distinguish between benign and malignant lesions, and some evidence suggests that it also plays a prognostic role in tumors that overexpress p16^INK4a^ [12].

p16^INK4a^ expression in RB remains controversial, especially in regarding of its expression patterns in histological phenotypes of RB [4,13,14]. Dimaras and colleagues reported that p16^INK4a^was expressed in fleurette regions but negative in undifferentiated regions [4]. Conversely, SE Coupland et al. described a reverse expression pattern of p16^INK4a^ that was positive in undifferentiated areas of 23 RB tumors [14]. To better understand p16^INK4a^expression and its potential role in RB, we evaluated the expression patterns of p16^INK4a^, Ki67 and CRX through a large cohort of 65 RB tumors. CRX is required for the terminal differentiation and maintenance of photoreceptors [15] and has been shown to be a differentiation marker of RB [16,17]. Our results demonstrated that p16^INK4a^ expression increased with the dedifferentiation of RB, showing strongly positive in undifferentiated cells and HWR, weakly to moderately positive in FWR, but negative in well-differentiated cells with fleurettes. This expression trend of p16^INK4a^ was consistent with that of Ki67, but reverse to that of CRX in RB tumors. The results indicated that p16^INK4a^ would be a valuable molecular marker of RB in distinguishing histological phenotypes and in serving as a predictor of its prognosis.

## Methods

### Tumor samples

Formalin-fixed, paraffin-embedded retinoblastoma blocks were retrieved from the archives of ocular pathology department at Zhongshan Ophthalmic Center of Sun Yat-sen University, China during a 3-year period (2008 to 2010). The cases with chemotherapy or radiotherapy prior to enucleation were excluded. Serial sections of 4 μm thick were cut, and subjected to hematoxylin and eosin (HE) staining. The tumor samples were divided into four groups according to the level of cell differentiation: undifferentiated (UD) group predominantly consisting of undifferentiated tumor cells and pseudorosettes; HWR or FWR group in which rosettes accounted for over 70% of the lesion according to the predominant rosette phenotype; and RC group in which fleurettes characteristic of photoreceptor differentiation accounted for over 60% of the lesion [1,2]. The study was approved by Medical Ethical Committee of Zhongshan Ophthalmic Center, Sun Yat-Sen University.

### Slide preparation and immunohistochemistry

Sections were deparaffinized in xylene, re-hydrated and incubated in 0.3% hydrogen peroxide (H_2_O_2_, Sigma, St. Louis, USA) for 30 min to block endogenous peroxidase activity. Antigen retrieval was achieved using 0.01 M citrate buffer, pH 6, in a pressure cooker for 45 s. For immunohistochemistry, the sections were stained with rabbit anti-CRX (1:200) (C7498, Sigma, St. Louis, USA), mouse anti-human p16^INK4a^ (ZJ11, Maixin-bio, China) and rabbit anti-Ki67 (1:200) (sc-15402, Santa Cruz, California, USA) respectively. Signals were detected using the Dako-Cytomation EnVision^+^ anti-rabbit or anti-mouse secondary system. The slides were then incubated in DAB solution (Vector Laboratories DAB substrate kit for peroxidase) for approximately 1 min, then washed in PBS and counterstained briefly in Harris hematoxylin. Images were taken using a BX51 microscope (Olympus, Japan). The primary antibody was replaced by dilution buffer as a negative control. The residual retinal tissue adjacent to tumor was considered as an internal control.

The sections were semi-quantitatively evaluated for the expression level of proteins according to reference with modifications [18]. Briefly, the intensity of CRX and p16^INK4a^ expression was scored on a scale of 0–2: 0, negative, 1, weak, and 2, strong; whereas the extent of both p16^INK4a^ and Ki67 expressions was scored as followings: grade 0, no staining; grade 1, < 40%; grade 2, 40-80%; and grade 3, 80–100% of tumor cells in the respective lesion. To analyze the association of p16^INK4a^ with clinicopathological features, both grade 0 and 1 were considered as low expression, both grade 2 and 3 as high expression. The high-risk feature evaluation included the presence and extent of optic nerve invasion and the massive choroidal invasion [19]. All specimens were screened and evaluated by two experienced pathologists blinded to the clinical information.

### Statistical methods

Spearman’s rank test and the Kruskal-Wallis test were used to analyze the progressive increase of p16^INK4a^ expression in various pathological types of RB. The association between the expression measured via immunohistochemistry and high-risk features was analyzed using chi-squared tests and Kruskal-Wallis tests. In all analyses, p < 0.05 was accepted as statistically significant. These analyses were performed using the SPSS 16.0 software package (SPSS Inc., Chicago, Illinois, USA).

## Results

### Clinicopathological features

Clinicopathological features were summarized in Table 1. Sixty-five enucleated eyeballs with RB were enrolled in this study. In our population, males (63.1%, 41/65) outnumbered females (36.9%, 24/65), with ages ranging from 1.73 months to 9 years and a mean value of 21.7 ± 20.0 months. The UD group accounted for 58.5% (38/65) of all tumors, and HWR 26.2% (17/65), FWR 10.8% (7/65) and RC 4.6% (3/65). Patients in the UD and RC groups were older than patients in the HWR and FWR groups, on average.

**Table 1:** **Clinicopathological features of RB tumors**

**Histopathological phenotype**	**Average age (m)**	**Gender**		**Eyes**		**Groups** ^**a**^			**High risk feature**
		**M**	**F**	**Unilateral**	**Bilateral**	**C**	**D**	**E**	**Number (%)**
UD	29.9 ± 21.8	22	16	35	3	8	16	14	14 (37)
HWR	9.4 ± 6.3	13	4	14	3	2	11	4	6 (35)
FWR	7.5 ± 6.9	5	2	4	3	2	3	2	1 (14)
RC	20 ± 13.9	1	2	2	1	2	1	0	0 (0)
Total	21.7 ± 20.0	41	24	55	10	14	31	20	21 (32)

^a^Grouping was according to international intraocular retinoblastoma classification (IIRC) [20].

### Expression patterns of p16^INK4a^ in retinoblastoma

**Figure 1:**
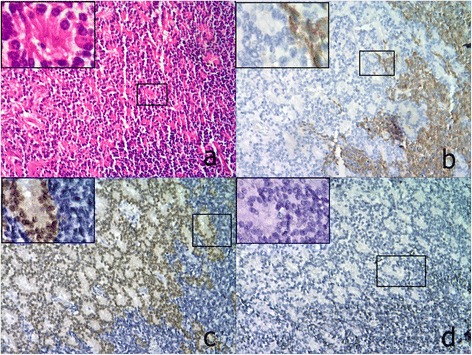
**Expressions of p16** ^**INK4a**^ **, CRX and Ki67 in a representative RB case from RC group (100x). a**. HE staining showed well-differentiated RC cells with typical fleurettes surrounded by moderately differentiated tumor cells; **b**. p16^INK4a^ was negative in the RC region, but positive in the surrounding tumor cells. A magnified image (insert) shows the transition zone between the RC and the tumor cells; **c**. CRX expression displayed the opposite pattern to that of p16^INK4a^, being strongly positive in RC region but negative in the surrounding area; **d**. Ki67 expression was similar to that of p16^INK4a^, with barely positive cells in RC region. All inserts, 200x.

**Figure 2:**
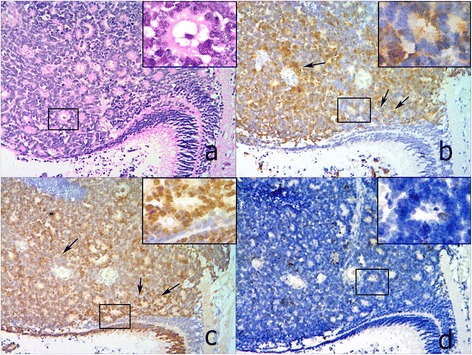
**Expressions of p16** ^**INK4a**^ **, CRX and Ki67 in a representative RB case from FWR group (100×). a**. HE staining showed FWR. **b**. p16^INK4a^ was moderately positive in FWR, but negative in the residual retina adjacent to the tumor. **c**. CRX expression was strongly positive in FWR, similar to the photoreceptors in the residual retina. **d**. Ki67 expression was similar to p16^INK4a^, with a few of positive cells scattered in FWR. When comparing b and c, a complementary expression pattern between p16^INK4a^ and CRX is seen (black arrow). All inserts, 200x.

**Figure 3:**
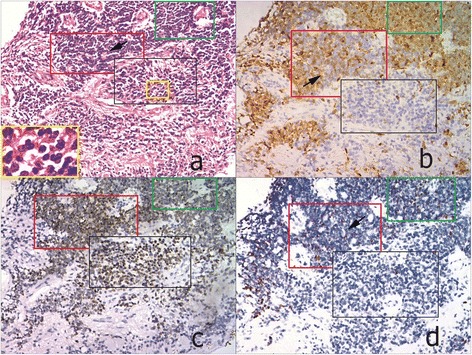
**Expressions of p16INK4a, CRX and Ki67 in different histological phenotypes of one RB case from HWR group (100×). a**. HE staining showed 3 histological phenotypes of RB, including RC (in black frame), HWR (in red frame) and UD (in green frame). The yellow frame highlighted fleurette structures in RC region with higher magnification (200x). **b**. The level of p16INK4a progressively increased from the region RC to HWR, then to UD. **c**. CRX was strongly positive in both RC and HWR region, but weakly to moderately positive in UD. **d**. The number of Ki67 positive cells scattered was increased from region RC, HWR to UD.

**Figure 4:**
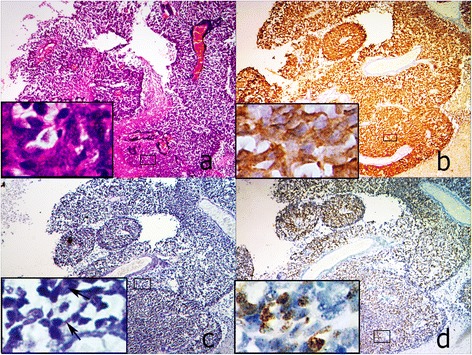
**Expressions of p16INK4a, CRX and Ki67 in a representative RB case from UD group (40×). a**. HE staining showed the tumor predominantly consisted of UD cells with scanty cytoplasm and large nucleus forming pseudorosettes. **b**. p16INK4a was strongly positive in both cytoplasm and nucleus of most of tumor cells. **c**. CRX expression was weakly to moderately positive in these tumor cells. **d**. Most of UD cells were positive for Ki67, similar to p16INK4a. All inserts, 200x.

Histologically, retinoblastoma consists of undifferentiated small round cells with scant cytoplasm and large nucleus. This tumor often contains certain amount of differentiated cells with more cytoplasm depending on the level of differentiation. The undifferentiated tumor cells usually scatter or form pseudorosettes around the blood vessel. In contrast, the differentiated cells can form specific structures from HWR or FWR to fleurettes with the increasing level of cell differentiation. In 65 cases, there were 3 tumors in RC that predominantly comprised of fleurette surrounded by some middle-size tumor cells. p16^INK4a^ in these fleurette structures was totally negative, which was similar to that in the residual adjacent retinal tissue, but positive in the surrounded tumor cells (Figure 1a, b). In FWR group, 4 out of 7 FWR weakly expressed p16^INK4a^, except for 3 cases (3/7) with moderate to strong immunoreactivities primarily located in the cytoplasm (Figure 2a,b). However, p16^INK4a^ expression in HWR area was similar to that observed in undifferentiated cells, most of which were strongly positive but primarily located in the cytoplasm (Figure 3a, b). Compared with the above groups, most of tumors (31/38) in the UD group were strongly positive for p16^INK4a^, which was located in both the cytoplasm and nucleus (Figure 4a, b), except for 5 cases (5/38) that were primarily cytoplasmic positive and 2 cases (2/38) that were weakly positive. As the degree of differentiation increased from the UD group to RC group, the expression level of p16^INK4a^ decreased (ρ = −0.770; p = 0.000; Spearman’s rank test).

### Correlation of p16^INK4a^ expression with the level of cell differentiation

To further determine the correlation of p16^INK4a^ expression with the level of cell differentiation, we detected the expression of both the photoreceptor differentiation marker CRX and the cell proliferation marker Ki67 in consecutive sections of retinoblastoma samples. In RC group, the fleurette strongly expressed CRX, but negatively Ki67 (Figure 1c, d), with the similar patterns to photoreceptor cells in the residual adjacent retina. In FWR region, cells were moderately to strongly positive for CRX with a few of Ki67 positive cells scattered (Figure 2c, d). In HWR region, cells were moderately positive for CRX with more Ki67 positive cells scattered (Figure 3c, d). Nevertheless, cells in UD region weakly to moderately expressed CRX with many Ki67 positive cells (Figure 4c, d). Therefore, considering together with the expression of p16^INK4a^ described above, our results demonstrated that p16^INK4a^ and Ki67 expression decreased, but CRX increased with the increasing level of cell differentiation. Using Spearman’s rank test, we found not only a positive correlation between p16^INK4a^ and Ki67 but also an inverse correlation between p16^INK4a^ and CRX, suggesting a possible relationship between p16^INK4a^ expression and the degree of RB differentiation.

### Correlation between p16^INK4a^ expression and clinicopathological parameters

The correlation was summarized in Table 2. Of 65 RB cases, 55 tumors (85%) had high expression of p16^INK4a^, and 10 tumors (15%) low expression. Most of tumors with high level of p16^INK4a^ expression originated from UD (36/55) and HWR (16/55) groups, whereas most tumors with low level of p16^INK4a^ from RC (3/10) and FWR (4/10). Statistic analysis showed the extent of p16^INK4a^ expression among these four groups existed significant differences (P = 0.000, Kruskal-Wallis test). In addition, tumors with high expression of p16^INK4a^ tended to have higher risk features with either optic nerve invasion (22%) or massive choroidal invasion (23%) even though statistically significant correlation was not found between the extent of p16^INK4a^ positivity and the presence of high-risk features. One of the two weakly positive p16^INK4a^ cases in the UD group had high-risk features that tumor cells were found in the line of surgical transaction. But no case with massive choroidal invasion had HWR, FWR or RC groups.

**Table 2:** **The relationship between the extent of p16** ^**INK4a**^ **expression and clinicopathological parameters**

	**P16** ^**INK4A**^		**P-value**
	**Low (0–1)** ^**f**^	**High (2–3)**	
Age (months)	13.6 ± 11.1	23.2 ± 20.9	P = 0.174^a^
Gender			
Male	6	35	P = 0.827^b^
Female	4	20	
Eyes			
Unilaternal	5	51	P = 0.000^b^
Bilaternal	5	4	
Differentiation^e^			
UD	2 (5%)	36 (95%)	P = 0.000^c^
HWR	1 (6%)	16 (94%)	
FWR	4 (57%)	3 (43%)	
RC	3 (100%)	0 (0%)	
High risk feature			
Optic nerve invasion	1 (10%)	14 (22%)	P = 0.676^b^
Massive choroidal invasion^d^	0 (0%)	15 (23%)	P = 0.100^b^

^a^Mann–Whitney rank sum test; ^b^Fisher’s exact test.^c^Kruskal-Wallis test. ^d^The criterion of massive choroidal invasion was the maximum diameter (thickness or width) equal to 3 mm or more in any diameter.^e^The number in brackets was the immunochemistry score of p16^INK4a^.

## Discussion

The development of RB involves sequential genetic lesions, and the loss of RB protein (pRB) is the first step and the basis of other biological alterations. In 1998, Schwartz found that pRB dysregulation resulted in increased p16^INK4a^ expression in colon cancer, due to positive feedback [21]. In recent years, the progressive increase of p16^INK4a^ expression accompanying pRB dysfunction had been described in many tumors [22]. In 2011, Witkiewicz summarized two complementary models for the appearance of tumors with high levels of p16^INK4a^ [6]. In the first model, p16^INK4a^ was elevated in some precancerous lesions due to oncogenic stress. If the RB1 gene was functional, the cells became senescent; otherwise, they became cancerous. In the second model, p16^INK4a^ increased via a feedback loop in response to pRB dysfunction and was associated with highly malignant tumors. The results of the present study were consistent with the second mechanism described by Witkiewicz.

Immunohistochemistry investigation of p16^INK4a^ in RB tumor samples had been reported with controversial results. Dimaras et al. described p16^INK4a^ expression in 8 RC cases, showing p16^INK4a^ positive in the benign RC region but negative in the RB area [4]. However, SE Coupland and colleagues reported the reverse expression pattern that p16^INK4a^ was negative in RC area but positive in poorly and moderately differentiated areas in their 23 RB tumors [14]. Similarly, Indovina et al. described that 6 out of 11 RB cases were 100% positive for p16^INK4a^ and that other cases were 5%-40% positive [13], implying that undifferentiated tumor cells were positive. In our 65 cases, the expression pattern of p16^INK4a^ in RB was closer to what SE Coupland described in a way that the level of p16^INK4a^ was increased from negative expression in well-differentiated fleurette structures to highly strong expression in poorly differentiated area. This pattern of p16^INK4a^ in RB was similar to that of Ki67, but reverse to that of CRX. Therefore, our study indicated that p16^INK4a^ could serve as a potential molecular marker to distinguish the level of cell differentiation in RB tumors.

The classic function attributed to p16^INK4a^ has been cell cycle regulation in the nucleus; however, p16^INK4a^ overexpression could occur in both the cytoplasm and nucleus of malignant cells. For example, in colorectal and breast cancers, p16^INK4a^ exhibited strong nuclear/cytoplasmic positivity in primary or metastatic carcinomas, whereas negativity or low nuclear expression was observed in normal mucosa and benign fibroadenoma [23,24]. Previous studies had shown that p16^INK4a^ appeared in the cytoplasm and/or nucleus in RB tumor cells [4,13,14]. Similarly, in our RB cases, the location of p16^INK4a^ expression largely depended on the degree of tumor differentiation, from staining in both the cytoplasm and nucleus of poorly differentiated cells to primary cytoplasm of moderately differentiated cells. Moreover, negative expression of p16^INK4a^ was seen in both the residual retina adjacent to the tumor and normal human retinal tissue. Nevertheless, the significance of different sublocations of p16^INK4a^ expression in malignant cells has not been clearly clarified yet,which may be associated with the prognosis of the malignant tumor. Many efforts have been made to understand the regulation mechanisms underlying p16^INK4a^ expression location within the tumor cells and its possible therapeutic value by re-locating the dislocated p16^INK4a^ to the optimal subcellular site in the malignancy [25-27]. More work is needed to answer these questions in the RB cases.

To date, the prognostic factors of RB remained a subject of intense discussion among ophthalmologists. The only consensus was that some high-risk histopathological features, such as the presence and extent of optic nerve invasion and the location and extent of uveal invasion [28], may be predictive. Grade of tumor differentiation has also been served as a key predictor of RB prognosis for a long time. In our 65 RB cases, both RC and FWR groups could be classified into the low grade of RB tumors, where most of tumors expressed low level of p16^INK4a^. However, the other two groups HWR and UD could be taken as the high grade of RBs, where most of tumors expressed high level of p16^INK4a^. Our results also demonstrated tumors with high expression of p16^INK4a^ had higher risk features with the optic nerve invasion and uveal invasion. These data suggested that the overexpression of p16^INK4a^ might be a risk predictor of the poor prognosis of RB tumors.

## Conclusions

In conclusion, our study demonstrates that p16^INK4a^ expression correlates with RB differentiation. The lack of p16^INK4a^ expression is a reliable marker of RC, a benign RB lesion. However, high-grade RB tumor is accompanied by an increase expression of p16^INK4a^, as determined by rigorous scoring approaches and multi-marker combinations. Our conclusions are based on a series of cases devoid of any follow-up information; thus, further investigation will be required to evaluate its value as a molecular marker of RB prognoses and therapeutic responses.

## Abbreviations

RB: Retinoblastoma
RC: Retinocytoma
CRX: Cone-rod homeobox
UD: Undifferentiated
HWR: Homer-Wright rosettes
FWR: Flexner-Winterstein rosettes

## References

[1] Kashyap S, Sethi S, Meel R, Pushker N, Sen S, Bajaj MS, Chandra M, Ghose S (2012). A histopathologic analysis of eyes primarily enucleated for advanced intraocular retinoblastoma from a developing country. Arch Pathol Lab Med.

[2] Suryawanshi P, Ramadwar M, Dikshit R, Kane SV, Kurkure P, Banavali S, Viswanathan S (2011). A study of pathologic risk factors in postchemoreduced, enucleated specimens of advanced retinoblastomas in a developing country. Arch Pathol Lab Med.

[3] Gallie BL, Ellsworth RM, Abramson DH, Phillips RA (1982). Retinoma: spontaneous regression of retinoblastoma or benign manifestation of the mutation?. Br J Cancer.

[4] Dimaras H, Khetan V, Halliday W, Orlic M, Prigoda NL, Piovesan B, Marrano P, Corson TW, Eagle RC, Squire JA, Gallie BL (2008). Loss of RB1 induces non-proliferative retinoma: increasing genomic instability correlates with progression to retinoblastoma. Hum Mol Genet.

[5] Eagle RC (2009). High-risk features and tumor differentiation in retinoblastoma: a retrospective histopathologic study. Arch Pathol Lab Med.

[6] Witkiewicz AK, Knudsen KE, Dicker AP, Knudsen ES (2011). The meaning of p16(ink4a) expression in tumors: functional significance, clinical associations and future developments. Cell Cycle.

[7] Courtois-Cox S, Genther Williams SM, Reczek EE, Johnson BW, McGillicuddy LT, Johannessen CM, Hollstein PE, MacCollin M, Cichowski K (2006). A negative feedback signaling network underlies oncogene-induced senescence. Cancer Cell.

[8] Hilliard NJKD, Sellheyer K (2009). p16 expression differentiates between desmoplastic Spitz nevus and desmoplastic melanoma. J Cutan Pathol.

[9] Lam AK, Ong K, Giv MJ, Ho YH (2008). p16 expression in colorectal adenocarcinoma: marker of aggressiveness and morphological types. Pathology.

[10] Garcia V, Silva J, Dominguez G, Garcia JM, Pena C, Rodriguez R, Provencio M, Espana P, Bonilla F (2004). Overexpression of p16INK4a correlates with high expression of p73 in breast carcinomas. Mutat Res.

[11] Ivanova TA, Golovina DA, Zavalishina LE, Volgareva GM, Katargin AN, Andreeva YY, Frank GA, Kisseljov FL, Kisseljova NP (2007). Up-regulation of expression and lack of 5’ CpG island hypermethylation of p16 INK4a in HPV-positive cervical carcinomas. BMC Cancer.

[12] Xu R, Wang F, Wu L, Wang J, Lu C (2013). A systematic review of hypermethylation of p16 gene in esophageal cancer. Cancer Biomark.

[13] Indovina P, Acquaviva A, De Falco G, Rizzo V, Onnis A, Luzzi A, Giorgi F, Hadjistilianou T, Toti P, Tomei V, Pentimalli F, Carugi A, Giordano A (2010). Downregulation and aberrant promoter methylation of p16INK4A: a possible novel heritable susceptibility marker to retinoblastoma. J Cell Physiol.

[14] Coupland SE, Bechrakis N, Schuler A, Anagnostopoulos I, Hummel M, Bornfeld N, Stein H (1998). Expression patterns of cyclin D1 and related proteins regulating G1-S phase transition in uveal melanoma and retinoblastoma. Br J Ophthalmol.

[15] Nishida A, Furukawa A, Koike C, Tano Y, Aizawa S, Matsuo I, Furukawa T (2003). Otx2 homeobox gene controls retinal photoreceptor cell fate and pineal gland development. Nat Neurosci.

[16] Glubrecht DD, Kim JH, Russell L, Bamforth JS, Godbout R (2009). Differential CRX and OTX2 expression in human retina and retinoblastoma. J Neurochem.

[17] Terry J, Calicchio ML, Rodriguez-Galindo C, Perez-Atayde AR (2012). Immunohistochemical expression of CRX in extracranial malignant small round cell tumors. Am J Surg Pathol.

[18] Zhao P, Hu YC, Talbot IC (2003). Expressing patterns of p16 and CDK4 correlated to prognosis in colorectal carcinoma. World J Gastroenterol.

[19] Canadian Retinoblastoma Society (2009). National retinoblastoma StrategyCanadian guidelines for care. Can J Ophthalmol.

[20] Linn Murphree A (2005). Intraocular retinoblastoma: the case for a new group classification. Ophthalmol Clin North Am.

[21] Schwartz B, Avivi-Green C, Polak-Charcon S (1998). Sodium butyrate induces retinoblastoma protein dephosphorylation, p16 expression and growth arrest of colon cancer cells. Mol Cell Biochem.

[22] Romagosa C, Simonetti S, Lopez-Vicente L, Mazo A, Lleonart ME, Castellvi J, Ramon y Cajal S (2011). p16(Ink4a) overexpression in cancer: a tumor suppressor gene associated with senescence and high-grade tumors. Oncogene.

[23] Di Vinci A, Perdelli L, Banelli B, Salvi S, Casciano I, Gelvi I, Allemanni G, Margallo E, Gatteschi B, Romani M (2005). p16(INK4a) promoter methylation and protein expression in breast fibroadenoma and carcinoma. Int J Cancer.

[24] Zhao P, Mao X, Talbot IC (2006). Aberrant cytological localization of p16 and CDK4 in colorectal epithelia in the normal adenoma carcinoma sequence. World J Gastroenterol.

[25] Souza-Rodrigues E, Estanyol JM, Friedrich-Heineken E, Olmedo E, Vera J, Canela N, Brun S, Agell N, Hubscher U, Bachs O, Jaumot M (2007). Proteomic analysis of p16ink4a-binding proteins. Proteomics.

[26] Shen WW, Wu J, Cai L, Liu BY, Gao Y, Chen GQ, Fu GH (2007). Expression of anion exchanger 1 sequestrates p16 in the cytoplasm in gastric and colonic adenocarcinoma. Neoplasia.

[27] Mills IG (2012). Nuclear translocation and functions of growth factor receptors. Semin Cell Dev Biol.

[28] Sastre X, Chantada GL, Doz F, Wilson MW, de Davila MT, Rodriguez-Galindo C, Chintagumpala M, Chevez-Barrios P, International Retinoblastoma Staging Working G (2009). Proceedings of the consensus meetings from the International Retinoblastoma Staging Working Group on the pathology guidelines for the examination of enucleated eyes and evaluation of prognostic risk factors in retinoblastoma. Arch Pathol Lab Med.

